# Longitudinal Effects of Activity-Based Flexible Office Design on Teamwork

**DOI:** 10.3389/fpsyg.2018.02016

**Published:** 2018-10-26

**Authors:** Christina Wohlers, Guido Hertel

**Affiliations:** ^1^DFG Research Training Group “Trust and Communication in a Digitized World”, University of Münster, Münster, Germany; ^2^Department of Psychology, University of Münster, Münster, Germany

**Keywords:** office design, activity-based flexible offices, new ways of working, teamwork, inter-team collaboration, longitudinal study

## Abstract

This three-wave longitudinal interview study (time lag: 12 and 18 months) investigates the impact of working in an activity-based flexible office (A-FO) on processes within and across teams (i.e., communication, trust, cohesion, and collaboration) and team management. Based on a new theoretical framework on benefits and risks of A-FOs (A-FO-M; Wohlers and Hertel, 2017), we conducted interviews with 25 employees of an in-house training institute who recently switched from single cell or shared offices to an A-FO. The A-FO consisted of a main open-layout environment without assigned workstations and provided additional working zones appropriate for specific work activities. According to the A-FO-M, A-FO features are expected to alter visibility and proximity of employees compared to office environments with assigned workstations. Altered visibility and proximity, in turn, should be related to team processes, such as communication. The interview material was analyzed using qualitative content analysis. This textual analysis procedure revealed that the interviewees reported that inter-team collaboration improved while working in the A-FO. Reasons that were mentioned for this positive effect were more contact, communication, collaboration possibilities (joint project work), and trusting relationships. However, interviewees also reported negative effects, such as that teamwork suffered due to less communication and cooperation. Along with that, especially ensuring team cohesion and communication among team partners were the most often mentioned challenges for management since team members were spatially dispersed within the office building. Theoretical and practical implications, such as assigning additional team areas to support teamwork, as well as recommendations for future research are discussed.

## Introduction

In the recent years, many organizations have adapted their office environments from traditional cellular or open-plan offices to more activity-based flexible office designs (A-FOs). These office designs consist of mainly open office environments, offer a variety of additional activity-related work areas, and make use of desk-sharing (e.g., De Been and Beijer, 2014; Hoendervanger et al., 2016; Wohlers et al., 2017). Despite spatial flexibility, also temporal flexibility with regard to work time hours is possible. Organizations often introduce A-FOs in order to save costs as well as to better respond to the requirements of knowledge work (i.e., to be able to concentrate on tasks as well as to share information with colleagues) compared to traditional office designs due to desk-sharing and provision of activity-based workspaces designed to support different activities (e.g., van der Voordt, 2004; Bodin Danielsson et al., 2014). Yet, adopting A-FOs also affects well-established structures and routines at work, for individuals as well as for teams and their supervisors, such as proximity and visibility of team partners.

While considerable research exists on the relation between physical elements of work environments and employees’ attitudes and behaviors (e.g., De Croon et al., 2005; Elsbach and Pratt, 2007; Davis et al., 2011), research has only started to address the effects of A-FOs on employees’ attitudes and behaviors. In particular, to the best of our knowledge, there are only a few studies investigating how A-FO designs affect teamwork (for notable exceptions see Volker and van der Voordt, 2005; Rolfö et al., 2017). None of these studies have focused on the management of teams except, although this is a critical factor of team success (e.g., Mesmer-Magnus et al., 2017). This is surprising, given the fact that teamwork is quite prevalent in organizations (Mathieu et al., 2014; Salas et al., 2015) and of critical importance for organizations’ success (Martin and Bal, 2006). Moreover, as conceptualized in the Activity-based Flexible Office Model (A-FO-M; Wohlers and Hertel, 2017), A-FOs are expected to alter proximity and visibility of team partners and non-team colleagues with important consequences for communication and collaboration among employees, which, in turn, are central for an effective organization (Wohlers and Hertel, 2017). Consequently, there is a need to empirically explore these relationships and their related consequences for teamwork in order to better understand the consequences of introducing an A-FO environment.

Therefore, in this article we examined how an A-FO design affects collaboration within and across teams. Based on the A-FO-M (Wohlers and Hertel, 2017), we particularly focused on aspects relevant for teamwork, such as information sharing, trust, and feeling of team cohesion (Mesmer-Magnus and DeChurch, 2009; De Jong et al., 2016; Mesmer-Magnus et al., 2017; Costa et al., 2018; Grossman and Feitosa, 2018) since these aspects ensure the functioning of teams and task accomplishment. Moreover, we considered team management as a critical aspect in A-FOs, including the motivation of team members, the support of team-building, and the prevention of coordination losses (e.g., Zaccaro et al., 2001; Boies et al., 2015; Aga et al., 2016). In doing so, this study also explored potential specific challenges for managers that might come along with the introduction of an A-FO. Finally, we explored for the first time whether the observed consequences of introducing an A-FO are stable over time, or only represent initial reactions toward a new office environment. In doing so, the current study focused on immediate reactions to the introduction of an A-FO, as well as potential changes across time. The study does not provide a pre-post assessment of collaboration outcomes before and after introducing an A-FO. However, capturing employees’ immediate reactions after the relocation provide important information about their subjective experiences and concerns, which are crucial in order to support the introduction of an A-FO and/or develop potential countermeasures if problems arise.

In doing so, we contribute to the literature in three ways: First, to the best of our knowledge this is the first study investigating how A-FO designs affect teamwork and inter-team collaboration. Filling this gap of knowledge provides valuable implications for the use of A-FO designs and on ways management should support teamwork and inter-team collaboration. Second, we also identify challenges for the management of teams that come along with an A-FO design, and derive practical implications to support organizations to adjust their policies and prepare supervisors and team members for the circumstances and tasks related to the introduction of A-FOs. Third, we investigate the effects of an A-FO design across time. In doing so, we provide information on whether (and which) immediate reactions after office redesign remain stable, or whether team members and supervisors adopt their routines and behaviors to the A-FO context.

### Activity-Based Flexible Offices Affect Working Conditions of Employees

Today’s knowledge workers perform a variety of different tasks with a varying degree of both, concentrated work alone or collaborative, interactive work with others, during a single workday (Hua et al., 2011; Davenport, 2013). These changing tasks pose constantly changing requirements to work environments to support the specific tasks. Office designs respond to these requirements with a varying degree of success (e.g., Davis et al., 2011). While cellular offices (i.e., private rooms that are enclosed by walls) are good in fostering undisturbed and private working and thus enable employees to concentrate on tasks, they lack to support communication and interaction among colleagues (Allen and Gerstberger, 1973; Kaarlela-Tuomaala et al., 2009). In contrast, open-plan offices (i.e., common-use workspace without barriers and enclosures hosting a larger group of employees whose desks are often arranged in groups) have the advantage of fostering communication and interaction, but have been shown to impede concentration of employees due to noise and uncontrollable interruptions by others (Brennan et al., 2002; Kaarlela-Tuomaala et al., 2009; Kim and de Dear, 2013).

Activity-based flexible office designs aim to respond to the whole range of work tasks. According to the definitions of Bodin Danielsson and Bodin (2008) and De Been and Beijer (2014), A-FOs consist of a main area in an open-plan layout and in addition provide a variety of open, half-open, or enclosed common used activity-related work areas. Examples are silent areas for concentrated work or project rooms that enable collaboration. To foster spontaneous and informal communication, A-FOs provide additional informal work areas, such as coffee lounges (De Been and Beijer, 2014). Another key feature of A-FOs is that employees have no assigned workstation (desk-sharing or hot-desking; Millward et al., 2007; Hirst, 2011) and choose workstations fitting to their current (task) needs. To support the easy switching of workstations, employees are equipped with portable computers and other information and communication technologies to be connected and available wherever they work. Besides the advantage to support a range of different work activities, A-FOs can often be dimensioned for <70% of the workforce based on expected illness and work outside the office (Duffy and Powell, 1997; Bodin Danielsson and Bodin, 2008).

Yet, besides the outlined benefits, A-FO features are expected to alter established routines and working conditions of employees, such as territoriality, autonomy, proximity, and visibility of employees, that are related to a variety of work-related outcomes at the individual, team, and organizational level (Wohlers and Hertel, 2017). Initial studies have already shown effects of A-FOs on job attitudes, such as job satisfaction (e.g., Appel-Meulenbroek et al., 2011; De Been and Beijer, 2014), well-being (e.g., Bodin Danielsson and Bodin, 2008; Bodin Danielsson et al., 2014), and performance indicators (e.g., De Been and Beijer, 2014). In particular, individual workers and also their communication and interaction in general have been considered. These studies yielded mixed results, i.e., there are studies describing an increase of communication (van der Voordt and van der Klooster, 2008; Blok et al., 2009, 2012) and others a decrease of communication (e.g., Rolfö et al., 2017). Beyond this, relatively little attention has been paid to effects of A-FOs on communication and collaboration when further distinguishing within and across teams, except for Volker and van der Voordt (2005) who’s case study revealed that the use of A-FOs was negatively related to social cohesion of team partners. Particularly, A-FOs’ features are expected to influence the visibility and proximity of team partners and non-team colleagues, which are strongly associated to the way how and where knowledge is shared and collaboration occurs (Appel-Meulenbroek et al., 2016). Hence, research on team-level effects within and across teams of A-FOs should be highly relevant for a complete understanding of the consequences of introducing A-FO designs in organizations.

### Teamwork and Inter-team Collaboration in Activity-Based Flexible Offices

The A-FO-M (Wohlers and Hertel, 2017) has conceptualized how both teamwork and inter-team collaboration may be affected when working in an A-FO compared to working in cellular or open-plan offices. Teamwork describes processes such as the interaction and relationship of team partners and their functioning as a team with respect to task coordination and accomplishment. Inter-team collaboration refers to the interaction and relationship between employees from different teams or departments. In contrast to general frameworks on team research, such as IPO or IMOI models (e.g., Ilgen et al., 2005), the A-FO-M explicitly models how crucial team processes are affected by the particular design and how these are related to important team outcomes. The general idea of the model is that A-FO-specific features influence working conditions of employees that in turn affect work-related consequences on the short and long term for individuals, teams, and the organization. According to this model, the interplay of central A-FO features, i.e., openness of main work environment, flexible use of activity-related work areas, and the use of desk-sharing, alters the fixed proximity and visibility of team partners and non-team colleagues compared to employees working in cellular and open-plan offices. Further, using desk-sharing, group boundaries as illustrated by the arrangement of workstations into clusters (in open-plan offices) or in a row (in cellular offices), are less salient in A-FOs. We expect that these altered working conditions affect crucial team-related processes, i.e., team cohesion, information sharing, and interpersonal trust, which are in turn related to team outcomes such as performance and satisfaction within teams, since these ensure the functioning of teams, task coordination, and accomplishment. More specifically, the A-FO-M postulates that teamwork in A-FOs is more difficult compared to in cellular and open-plan offices, where team partners sit relatively close to each other. In A-FOs, employees can choose a workstation in a variety of different work areas. Consequently, team partners can be spread all over the office building. In addition, most organizations using A-FOs also allow employees to work outside the office building, which negatively affects the proximity and visibility of team partners even more. Proximity and visibility, however, have been shown to positively influence information sharing (e.g., Appel-Meulenbroek et al., 2016), feelings of group cohesion (e.g., Brennan et al., 2002), and also to foster the development of trust among team partners (e.g., Lewicki and Bunker, 1995; Mesmer-Magnus and DeChurch, 2009) – highly important team processes positively influencing team satisfaction and team performance. Especially interpersonal trust might become particularly important in A-FOs, since team members of interdependent work groups have to cope with situations of high uncertainty. For example, due to low proximity and visibility, they cannot see their team partners working, and trust can help to overcome such situations entailing uncertainty (Jarvenpaa et al., 1998). Yet, low proximity and visibility of team members in A-FOs might even hinder the development of trust since cues about the trustworthiness of a team partner (ability, integrity, and benevolence; e.g., Mayer et al., 1995) are more difficult to gather when team members do not work in sight of each other. Together, we expect that teamwork is negatively affected by the A-FO design, which should be manifested in less personal contact among team partners, less information sharing, lower feelings of team cohesion, and lower ratings of interpersonal trust compared to team partners working in cellular or open-plan offices.

In contrast, collaboration across team boundaries should be facilitated in A-FOs according to the A-FO-M, because employees often work closer to non-team colleagues in A-FOs compared to cellular or open-plan offices. Further, the openness of the main work environment and the offer of activity-related work areas designed to prompt spontaneous interaction and informal communication (e.g., coffee lounge, table soccer) are supposed to stimulate interaction and communication among non-team colleagues. Thus, we expect that A-FOs have a beneficial effect on collaboration across existing teams or work units as measured by an increase of contacts across teams, establishment of trusting relationships, and joint project work.

### Managing Teams in Activity-Based Flexible Offices

In addition to the aforementioned critical team success factors from the A-FO-M, leadership is another critical factor influencing the effective functioning of teams (Zaccaro et al., 2001). In fact, team leadership or management is a key mechanism maintaining team performance by reducing motivation and coordination losses in teams, especially when teams are virtual (Zigurs, 2003; Malhotra et al., 2007). Yet, considerable research on virtual teams has demonstrated that traditional hierarchical leadership behaviors, such as motivating team members and managing team dynamics, are more intricate in virtual teams because of a lack of face-to-face contact, often asynchronous communication and geographical distribution of team members and thus should be supplemented to maintain team performance (e.g., Avolio et al., 2014; Hoch and Kozlowski, 2014). Hierarchical leadership behaviors are addressed in prominent leadership theories, such as, for example, leader-member exchange (LMX, Graen and Uhl-Bien, 1995; Gerstner and Day, 1997) or transformational leadership (Bass, 1985, 1998; Bass and Riggio, 2006). Both LMX and transformational leadership have been shown to considerably impact team performance of traditional and virtual teams (e.g., Howell et al., 2005). However, their impact on virtual teams is weaker because leadership behaviors are more ambiguous and difficult to interpret when face-to-face contact with the leader is scarce. In addition, the nature and quality of leader-member relationships primarily develop through face-to-face contact (Gerstner and Day, 1997). Thus, they are harder to develop when the leader seldom has face-to-face contact to followers. Different ways of supporting virtual leadership to overcome these difficulties have been discussed, among them augmenting leadership by the use of electronic communication media (Avolio and Kahai, 2003), distributing leadership to team members (shared leadership), as well as structural supports (Bell and Kozlowski, 2002; Hoch and Kozlowski, 2014). Still, these approaches have been shown to be time intensive and cost a lot of energy (Purvanova and Bono, 2009).

Yet, given the fact that A-FOs are expected to alter the proximity and visibility of team members, also the way leaders can take influence on them might be impacted, since team members are no longer grouped into clusters as they used to be in traditional cellular or open-plan offices. Furthermore, we assume that also the distance between leaders and employees is affected by the A-FO design, since employees can be spread within the office, making it harder to be seen and found by leaders. Accordingly, the team management in A-FOs seems to partly resemble the management of virtual teams regarding the reduced proximity and visibility of team members and leaders. Still, it is much easier to meet each other and arrange face-to-face team meetings in A-FOs compared to virtual teams, since leaders and team members often are in the same building. Hence, it is interesting to explore challenges for team leadership when switching from traditional offices to A-FOs. In line with the aforementioned arguments, it seems plausible that leaders have to find ways to adopt their leadership behaviors to the A-FO context in order to ensure the functioning of teams and thus remain effective. Therefore, this study particularly addresses challenges for leadership arising in the context of A-FOs and derives ways to support teams in A-FOs.

### A-FO Effects Over the Course of Time

In the current study, we investigate effects of an A-FO design on working conditions with respect to teamwork and management over the course of time. In fact, this is one of the first empirical studies addressing such longitudinal effects of A-FOs on employees’ attitudes, well-being, and behavior (for exceptions, see Gerdenitsch et al., 2017; Rolfö, 2018). Most existing studies have been conducted right after relocation to the new environment (e.g., Blok et al., 2009; Appel-Meulenbroek et al., 2011; Rolfö et al., 2017; Candido et al., 2018) and therefore focused mainly on immediate reactions toward the change of working conditions. In contrast, longitudinal studies can investigate employees’ reactions toward the A-FO at different phases of the implementation process in order to identify first excitement toward the new situation (“honeymoon effect”) and later various adaptions to the new working conditions (e.g., Singer and Willett, 2003; Ployhart and Vandenberg, 2010). That way, the long-term stability of effects can be identified. In particular, we were interested if initial reactions to a new A-FO design change over time starting from first excitements to the establishment of new routines or ways to cope with the new working conditions, which is in line with change management models describing people’s psychological reactions over time toward a change process, such as the Kübler-Ross model (Kübler-Ross, 1969). Thus, we investigated whether reactions to a new A-FO are stable, or whether they change over the course of time. Further, certain team processes need time to develop especially when team partners are physically dispersed, such as trust development (Wilson et al., 2006; Grossman and Feitosa, 2018). Hence, possible effects of A-FOs on trust can only be observed after working in an A-FO for a longer period, implying the need for longitudinal study designs.

To summarize, the current exploratory study aims to shed further light on short- and long-term A-FO effects on intra- and inter-team processes and team management by addressing the following research questions: How does the A-FO design affect team processes, such as trust and information sharing, within teams? How is collaboration across teams affected by the design? Finally, what are the challenges for the management of teams in A-FOs? For each question, we also want to address the stability of the effects over time.

## Methods

### Research Setting

This longitudinal interview study was conducted with employees of an in-house training institute from a globally active engineering company. The training institute consisted of 136 office workers and was responsible for the development, execution, and sale of in-house HR trainings, such as cultural competence training. The training institute was clustered into seven functional departments, four of which were subdivided into three to four teams each. The teams of each department were managed by a first-level supervisor. The teams worked jointly for the goals of the department but had clear functional responsibilities, for which they worked autonomously. Each team consisted of five to 19 team members and was led by a team manager. Team members had unique responsibilities but worked interdependently, i.e., they had to cooperate and work interactively to reach their common goals. They engaged primarily in cognitive tasks, such as thematic development of training units, project management or operative tasks, such as acquisition, support, and operation processes.

The redesign of the office environment of the training institute was part of a global change strategy of the organization. The main goals of the redesign that were communicated by the organization were to create attractive work environments to attract and bind talented employees, and to foster creativity and performance of employees. In addition, the redesign should encourage communication and new ways of collaboration.

The implementation of the new office followed the global rollout-guidelines of the organization, including typical change management strategies such as a project group with stakeholders from different teams, workshops with managers as role models, communication of goals and progress of change initiatives (e.g., via newsletters), and active involvement of employees in work groups preparing the new office (e.g., for designing and naming workspaces).

Prior to switching to the new office environment, all employees had assigned workstations and mostly worked in single cellular office rooms or shared an office with one to three colleagues on the same floor. Supervisors each had a single office. The head of the training institute and his/her assistances were located on another floor. All tasks were mainly done at individual workstations, including writing, conceptual work, phone calls, and conversations with colleagues.

The new office environment was realized in a new building in another part of the city, where already other sectors and departments of this organization were located. It consisted of a large main area in an open layout, where most of the workstations were available, including standard workstations and “hot desks” (space useable for shorter periods). In addition to the main area, there were four groups of functional work areas (i.e., concentration, creativity, communication, and service), which were designed to support employees’ needs for concentrated work or collaborative work with others. Work areas supporting concentration are, for example, so-called silent areas, where all kinds of disturbances (i.e., noise and disruptions) are forbidden. Creativity of employees should be fostered, for example, by creativity rooms, which offer space for three to eight employees and provide unconventional, inspirational furnishings in contrast to the standard workstations. Formal (planned) as well as informal (coincidental) communication is enabled by phone boxes, meeting rooms, or a meet-and-talk area. The last group of work areas was service facilities, such as lockers for employees, postboxes, or a centrally located copy and print area. The furniture was modern and colorful and built with materials that can buffer noise. For every workstation, employees were asked to clean their desks when leaving so that it is free for the next user (clean-desk policy). A list and description of the work areas and their purpose is provided in the Supplementary Material. Guidelines for the effective use of these different work areas were communicated to the employees. Almost all employees had non-assigned workstations, except the employees of the IT service unit and the senior manager of the training institute. The employees of the IT service unit shared an office and the senior manager had her/his individual room, which could be used for other purposes when she/he was not present.

### Design

We conducted a three-wave longitudinal interview study with 26 semi-structured interviews with an approximate length of about 45 min each (min: 27, max: 68 min) over a period of 2.5 years. Since team effects in A-FOs have not received much attention in research and theory so far, it is not possible to reliably derive testable hypotheses. Therefore, we chose interviews as an appropriate method to explore this new topic. Further, the interviews were semi-structured in order to allow aspects that are of importance for the interview partner to be mentioned and further discussed and still enable a comparison between participants (Rapley, 2004). All interviews were conducted by the first author in order to diminish possible interviewer effects across persons and waves. That way, we made sure that all interviews were done in the same way. To diminish person-related artifacts, the interviewer trained using the interview protocol with five colleagues from the university department prior to the interviews. The first wave of interviews with 19 employees and 7 supervisors was conducted 4 weeks after relocation to an A-FO as data collection was not possible prior to the relocation. The second wave took place 12 months after relocation. The third wave took place 30 months after relocation (18 months after the second wave).

The first wave of interviews examined immediate reactions toward the new A-FO design, for instance, capturing initial excitements about the new work environment (“honeymoon-effect”). The second and third wave examined the stability of such effects. In general, it is recommended to include more than two waves, in order to also capture non-linear changes (Singer and Willett, 2003). The time lags between the waves were selected according to models describing reactions toward change (e.g., Kübler-Ross curve; Kübler-Ross, 1969), the fact that some constructs of interest show a very slow evolution over time (e.g., trust and team cohesion; Wilson et al., 2006), and the availability of the corporation partner. In general, we relied on self-reported data, which can be sufficient when research is focusing on subjective experiences and perceptions (e.g., Chan, 2009; Conway and Lance, 2010). All interviews were audiotaped, except for two cases, where participants refused to be recorded. In these cases, paper–pencil notes were made during the interview. The audiotaped interviews from the first wave were transcribed verbatim. Given that the coding scheme had already been established on the basis of the first wave material, we waived to transcribe the audio material verbatim from the second and third wave and instead made notes from the audio material as a help for the coding procedure described in the section “Data Analysis”. Intensive effort spent on a further verbatim transcription would not have led to additional information.

The interview questions covered the experienced benefits and risks of working in A-FOs with respect to a range of different aspects (workplace flexibility, autonomy, teamwork, leadership, motivation, satisfaction). The questions were derived in an inductive way, based on the main constructs and relationships modeled in the A-FO-M by Wohlers and Hertel (2017). In the current paper, we only focus on the interview questions that are relevant for teamwork, collaboration across teams, and management of teams. The interview protocol was structured as follows: We started the interviews with asking employees to describe their jobs, their organizational position, and their current work duties followed by asking participants to explain how the new office environment affects different aspects of work. These aspects included: teamwork within and across teams in general, team dynamics, and processes such as communication, trust, cohesion, and team outcomes such as collaboration, as well as challenges for the management of teams. Note that while at the first wave only supervisors were asked to explain how team management is affected, at the second and third also employees’ perspective on challenges for team management was assessed. This was due to supervisors suggesting during the first wave that the employees’ perspectives would be interesting as well, because these would directly experience possible adaptions in the leadership behavior in the A-FO context. Finally, a range of demographic information (gender, age, status, organizational tenure) was also gathered. Following guidelines for conducting interviews (e.g., Jacob and Furgerson, 2012; Rowley, 2012), the structured interview protocol first asked more general open-ended questions (e.g., “Please describe how you currently communicate with team partners/non-team colleagues/supervisors”), followed by more specific probe questions (e.g., “How did communication change since working in the A-FO and whereby?”; “How is informal/formal communication affected in the A-FO?”) for each topic. To control for any influences of unrelated factors, the interviewer always asked if reported effects of the new office design were truly related to the office design, or to external influences. Only those observations that were clearly related to the new office design were coded and further analyzed in this study.

### Sample and Procedure

An employee from the organization arranged the interviews with the participants. Employees out of one of three groups who recently participated in an in-house training were asked to take part in our study. All subjects participated voluntarily in the interviews in accordance with the Declaration of Helsinki and no incentives were provided for participation. The participants were informed about the aims of the study and about the possibility to withdraw at any time or skip any parts of the interviews without reprisal. Audio recordings of the interviews were only conducted with explicit approval of the participants. The data recorded during the interviews was stored and analyzed ensuring the anonymity and privacy of the participants. No ethical review or approval was required for this study under the national or international requirements. This study adheres to the recommendations of the Federation of the German Psychologists Association’s Code of Ethics. Out of our total sample (*N* = 28), we were able to interview 26 employees from different teams twice and 15 three times. Reasons for dropouts were: partial retirement (3), in-house job transition (1), heavy workload (3), extern job transition (3), and two persons refused the interviews. We excluded the data of the head of the training institute as he was not involved in teamwork. We could match the data of *N* = 25 participants (19 employees, 6 supervisors) for the first and second wave. A total of *n* = 14 employees (10 employees, 4 supervisors) participated in all three waves. Among the 25 (14) participants, 11 (8) were male and 14 (6) female with a mean age at the first measurement time of 45 years (*SD* = 8.81; age range = 30–58 years) and *M* = 11.25 years (*SD* = 6.40) of organizational tenure. Supervisors had a mean manager-to-staff ratio of *M* = 12.37 employees (*SD* = 5.54; range: 5–19 employees) and were responsible for one to four teams. Table 1 gives an overview of the composition of the sample at each measurement time.

**Table 1:** Overview of the sample.

Composition of the sample	Time 1 (4 weeks after relocation)	Time 2 (12 months after relocation)	Time 3 (30 months after relocation)
*N*	25	25	14
Employees	19	19	10
Supervisors	6	6	4
Male	11	11	8
Female	14	14	6
Mean age (in years)	44.74 (*SD* = 8.81)	45.46 *(SD = * 8.83)	47.43 (*SD* = 5.33)
Age range	30–58	31–59	39–58
Organizational tenure (in years)	11.25 (*SD* = 6.40)	12.10 (*SD* = 6.44)	14.7010 (*SD* = 4.87)

### Data Analysis

The interview material was analyzed with an iterative and inductive textual analysis according to the guidelines of qualitative content analysis introduced by Mayring (2010, 2014). The first author and a research assistant started with building broad categories by first reading the material multiple times and making notes and comments in the transcripts that seemed relevant for the research questions (e.g., reported risks for teamwork within teams, changes in communication behavior). This way, the general topics this study focuses on were determined. Basing on these notes and comments, the text was divided into thematic units. These units were then organized into conceptually similar categories (e.g., risk for teamwork because of hampered communication and interaction, risk of teamwork because of reduced visibility and proximity). In addition, we aggregated quotes along the relevant categories in order to describe and label them. After this procedure, we discussed and agreed on the final set of main- and subcategories, their (re-)definition, and coding rules. After having set the final coding scheme, two independent coders (i.e., blind to the motivation of the study) coded all transcripts, audio-material, and notes. Most of the categories were dichotomous and the coders had to judge whether a specific category was mentioned in the interview or not, which made the information countable and allowed us to calculate frequencies. Both coders first practiced coding the interview material by test coding five interviews. In case of non-agreement, the coders discussed the categories and adjusted the coding rules. After this test procedure, the coders coded all the material for the three waves. Cohen’s kappa as an estimate of reliability (agreement between the two raters) was *κ* = 0.70. In order to improve the coding, coders discussed crucial categories in which inter-reliability was low and coded these specific categories again. Cohen’s kappa after discussion was *κ* = 0.83, which can be considered as satisfying (Landis and Koch, 1977).

## Results

Overall, the interviewees reported that the A-FO design affected teamwork and collaboration across teams. In addition, several challenges for team management were mentioned by the interviewees. Table 2 summarizes the frequencies of identified categories regarding teamwork, inter-team collaboration, and team management. We present these findings in three sections. First, we describe the identified categories that are related to teamwork, followed by those related to inter-team collaboration, and finally, we describe the categories that are related to challenges for the management of teams. Note that the frequencies that are reported in this study always refer to the group of interviewees that made a statement by themselves matching a certain category, e.g., 41.7% of the interviewees reported a decrease of teamwork. This does not directly imply that the rest of the interviewees reported something opposite, e.g., 58.3% could have either reported an increase of teamwork, no change of teamwork, or did not make any statement matching these categories. The quotes presented in the section “Results” are chosen to show representative statements for the categories. Quotes will not be compared across time since they represent examples for the categories, which are already counted and compared across time.

**Table 2:** Frequencies in percent of teamwork, inter-team collaboration, and team management categories.

Category	Time 1 (*n* = 25)	Time 2 (*n* = 25)	Time 3 (*n* = 14)
**Decline of teamwork**	41.7	33.3	50.0
Physical distance and visibility of team partners	16.7	28.0	50.0
Hampered communication and interaction	41.7	33.3	50.0
Low levels of personal communication	25.0	32.0	28.6
High levels of digital communication	33.3	56.0	57.1
Hampered cooperation among team partners	16.7	25.0	28.6
Impeded trust among team partners	4.0	4.0	14.3
**Increase of inter-team collaboration**	68.2	66.7	71.4
Physical closeness and visibility of non-team colleagues	27.8	32.0	35.7
Eased communication among colleagues	63.6	66.7	64.3
New contacts across teams	54.5	45.8	57.1
Joint project work across teams	13.6	20.8	28.6
Trusting relationships with non-team colleagues	20.8	16.0	21.4
**Challenges of leadership in regard to management of team members^1^**			
Managing physically dispersed team members	66.7	64	85.7
Hampered communication	50	32	35.7
Stabilizing the functioning of teams	50	44	42.9
Demand of coordination and time management skills	33.3	36	42.9

*The comparison with time 3 frequencies is limited in validity as the sample size is reduced. ^1^At time 1 only the supervisors (n = 6) were asked for challenges of team management. At time 2 and 3 both, employees and supervisors were asked for this topic. The sample of time 2 consists of 19 employees and 6 supervisors and the sample at time 3 consists of 10 employees and 4 supervisors.*

### Teamwork Within Teams

The interviewed persons described various challenges of the A-FO design for processes within the existing teams. At *t*_1_, 41.7% of the interviewees reported that teamwork declined when working in the A-FO. This percentage slightly decreased at *t*_2_ (33.3%) and increased again at *t*_3_ (50.0%). The interviewees reported several reasons for the experienced negative impact of the A-FO on teamwork:

*Physical distance and visibility of team partners*. According to the interviewees, the use of desk-sharing and the availability of different work areas affected physical distance among team partners. In the A-FO, team partners were not located close to each other anymore, but instead they were spread within the office building and thus were less visible to each other. At *t*_1_, 16.7% of the interviewees reported a reduced visibility of their team partners because they were spread all around the office building or even worked outside the office building. This percentage further increased at *t*_2_ (28.0%) and at *t*_3_ (50.0%). Thus, visibility of team partners was reported to be declined during working in the A-FO.

As noted by the interviewees, the physical distance and low visibility of team partners in turn were made responsible for a negative influence on a variety of team-related processes and conditions relevant for teamwork (i.e., communication and interaction, type of communication, cooperation, and trust), which we discuss in the following.

*Hampered communication and interaction*. At *t*_1_, 41.7% of the interviewees complained about reduced personal contact and communication frequency within the teams after the A-FO redesign. More specifically, interviewees reported that due to the physical distance among team partners, conversations, agreements, and task coordination were hampered. This percentage slightly decreased at *t*_2_ (33.3%) and increased again at *t*_3_ (50.0%). For instance, one interviewee stated:

“I have realized that in my team we often didn’t know what the other person is doing. Information exchange isn’t like it used to be (anymore).”

(Interviewee number 13, male, supervisor, *t*_1_)

*Amount of digital vs. face-to-face communication*. At *t*_1_, 25.0% of the interviewed persons reported that personal, face-to-face communication decreased after switching to the A-FO. This decrease endured during *t*_2_ (32.0%) and *t*_3_ (28.6%). Nevertheless, even though personal communication and visibility were perceived to be reduced in A-FOs compared to the previous office environment, accessibility of team partners was still described as good. One interviewee reported:

“Accessibility hasn’t changed. We have telephone and email. Visibility is reduced, even though this is an open office.”

(Interviewee number 21, male, employee, *t*_3_)

This quote illustrates that digital communication enabled team partners to still be connected with each other, even though they see each other less often face-to-face. In line with this, at *t*_1_, 33.3% of the interviewees reported that computer-mediated communication increased during working in the A-FO. This percentage further increased at *t*_2_ (56.0%) and at *t*_3_ (57.1%). Thus, it seemed that instead of searching for team partners and meeting face-to-face within the office building, team members made use of electronic communication media, as illustrated by the following response of one interviewee:

“I write a lot more emails than in the past. This is because in the old office, I just went over into the next office to talk. And now … this doesn’t work anymore since I don’t know where the team partners are. This is why I now use emails for all that [communication].”

(Interviewee number 23, female, supervisor, *t*_1_)

*Hampered cooperation among team partners*. In addition, interviewees reported that they perceived that working in the A-FO negatively affected cooperation among team partners. At *t*_1_, 16.7% of the interviewees reported that cooperation among team partners, such as working together on a task or helping each other, suffered due to the A-FO design. These complaints slightly increased during the course of the interview study (*t*_1_: 16.7%; *t*_2_: 25.0%; *t*_3_: 28.6%). For example, one interviewee commented:

“The main alteration indeed is that people who work on the same tasks don’t exchange information as much as necessary to prevent duplication of effort. This means that work is more inefficient since communication is disrupted. This is mainly due to the fact that it takes a lot more effort for communication to take place – a room needs to be found and everyone has to be informed where this room is to get together.”

(Interviewee number 13, male, supervisor, *t*_2_)

*Impeded trust among team partners*. Regarding trust among team partners, the interviewed persons described no strong impact of A-FOs on trust among team partners, i.e., the intention to make oneself vulnerable to the actions of the team partners. A large amount of the employees reported that trust in general (without further mentioning the trustor and trustee) was affected by the A-FO design (*t*_1_: 50.0%, *t*_2_: 32.0%, *t*_3_: 64.3%). After asking to further specify the trust relationships, however, only a few interviewees, namely one (4.0%) at *t*_1_ and one (4.0%) at *t*_2_ mentioned that trust among team partners was negatively affected by the A-FO design, only exhibiting a slight increase to *t*_3_ (14.3%). Thus, interviewees did not report that trust among team partners was negatively affected when working in an A-FO. Please see also the results on trusting relationships with non-team colleagues in the section “Collaboration across Teams (Inter-team Collaboration)” for other categories of trust mentioned.

Overall, the interview data suggested that A-FO features rather hindered communication and collaboration among team partners due to physical distance among team partners. Interviewees reported that when team partners were spread within the office building, they were not visible to each other. Consequently, they communicated more often via electronic media instead of talking face-to-face. Surprisingly, trust among team partners was not reported to be affected during working in the A-FO design.

### Collaboration Across Teams (Inter-team Collaboration)

In contrast to the reported risks for teamwork, interviewees reported that they experienced that collaboration across teams improved while working in the A-FO design (*t*_1_: 63.6%, *t*_2_: 66.7%, *t*_3_: 71.4%). The interviewees mentioned several possible reasons for the A-FO’s positive impact on collaboration across teams that can be summarized into the following categories:

*Physical closeness and visibility of non-team colleagues*. At *t*_1_, 27.8% of the interviewees reported that, in contrast to the previous office environment, non-team colleagues sat closer to each other and were more visible to each other due to the use of desk-sharing and the open character of the main office area. This increase endured during *t*_2_ (32.0%) and *t*_3_ (35.7%).

*Better communication among non-team colleagues*. At *t*_1_, 63.6% of the interviewees reported that they communicated more often face-to-face with non-team colleagues since working in the A-FO context. This tendency endured over all three measurement times (*t*_1_: 63.6%, *t*_2_: 66.7%, *t*_3_: 64.3%). For example, one supervisor commented:

“Across teams, a lot has changed informally. Since I see my colleagues and spend days next to them at the desk, there is an exchange of information. ‘What are you doing, what am I doing? Oh, I can help!’ This way, exchanging information is much quicker. This is a real positive effect.”

(Interviewee number 26, female, supervisor, *t*_3_)

However, having a closer look at the nature of inter-team communication, interviewees reported that informal communication, i.e., communicating about personal, non-work-related issues increased stronger (*t*_1_: 50.0%, *t*_2_: 44.0%, *t*_3_: 78.6%) compared to formal communication, i.e., communication about work-related issues (*t*_1_: 12.5%, *t*_2_: 40%, *t*_3_: 14.3%), when working in the A-FO. One interviewee reported:

“I do small talk more than ever before. In other words, I talk about my vacation with the people, about the weather, about technical stuff. On the next day, a new person is sitting next to me and I talk about these topics again. Again and again. That gets on my nerves. I do small talk non-stop but this doesn’t produce anything useful.”

(Interviewee number 23, female, supervisor, *t*_1_)

*New contacts across teams*. Interviewees stated that they could easily establish more contacts across teams in the A-FO context (*t*_1_: 54.5%, *t*_2_: 45.8%, *t*_3_: 57.1%). This might be due to non-team colleagues being more visible in the A-FO design compared to the old office environment, as mentioned by one-third of the interviewees (*t*_1_: 27.8%, *t*_2_: 32.0%, *t*_3_: 35.7%):

“Another alteration is the visibility of non-team colleagues. In the past, there were no contact areas, no topics to talk about. I knew who they were, but we had no contact at all, no lunches together … nothing … Nowadays, this is totally different. I see these guys more often and have contact.”

(Interviewee number 2, male, employee, *t*_2_)

Establishing more contacts across teams might have been beneficial for joint project work across teams, as illustrated by the following example:

“Before, when I had to make an offer, I could only ask one expert. Now I can ask two or three more experts from different departments since I got to know them and know what they are working on.”

(Interviewee number 2, male, employee, *t*_2_)

*Joint project work across teams*. The tendency toward joint project work between existing teams was described to slowly increase after the A-FO redesign (*t*_1_: 13.6%, *t*_2_: 20.8%, *t*_3_: 28.6%). Although there was a benefit for establishing inter-team contacts and communication, surprisingly few employees have mentioned that joint project work across teams benefited from the A-FO design. The tendency toward such joint project work, however, was perceived as very positive by the interviewees and they reported that they would enjoy a further increase of it.

“Currently, I am working on single projects with colleagues from other departments. Collaboration across teams [in form of joint projects] indeed has increased due to the open structure, but in my eyes this is not as strong as it could and should be.”

(Interviewee number 9, male, employee, *t*_3_)

Along with that, one supervisor outlined that joint project work might need to be especially supported:

“We often think about ways how to foster collaboration across teams [joint projects]. In my eyes this is important since that way we can create synergies and reduce lack of resources. Until now we don’t have an incentive system for collaboration across teams. Employees should be rewarded for collaboration across teams so that it counts the same way as it would when working for the own team. That way they do not always have to balance how much effort they spend for other departments or whether they should better spend their time for their team.”

(Interviewee number 26, female, supervisor, *t*_3_)

*Trusting relationships with non-team colleagues*. Finally, employees reported that they established more trusting relationships to non-team colleagues (*t*_1_: 20.8%, *t*_2_: 16.0%, *t*_3_: 21.4%). According to the interviewees, the reduced distance to non-team colleagues supported the establishment of trusting relationships as this facilitated getting familiar with non-team colleagues as described by this interviewee:

“Also, trust towards other business units has strongly increased because we know each other, go for lunch together or talk to each other. This is really strong.”

(Interviewee number 2, male, employee, *t*_3_)

Overall, the interview data suggested that A-FO features, in particular the use of desk-sharing and openness of the work environment, positively influenced the development of contacts as well communication across teams due to the physical closeness and visibility of non-team colleagues. In addition, working in the A-FO facilitated getting familiar with non-team colleagues and in turn supported establishing trusting relationships. However, although interviewees reported a benefit for inter-team collaboration regarding contact, communication, and trust, only three to five interviewees reported an increase of joint project work across existing teams.

### Challenges for the Management of Teams

In addition to the implications of the A-FO on teamwork, the interviewees stated that the A-FO design critically affected supervisor–follower relationships, in particular with respect to the managing of teams. Table 2 gives an overview of challenges for team management that, according to the interviewees, arose while working in the A-FO design.

*Managing teams with physically dispersed employees*. Supervisors and employees consistently reported challenges for the management of team members physically spread within or even outside the office building, which seemed to increase while working in the A-FO (*t*_1_: 66.7%, *t*_2_: 64.0%, *t*_3_: 85.7%). Often, interviewees compared the management of teams in A-FOs to the management of virtual teams, as team members worked out of sight of supervisors, as mentioned by one interviewee:

“Work has become more virtual. Not completely, as we are still able to meet each other in person. Beforehand, I just walked over to my employees and gave them the required information. Now I don’t do that anymore. Instead, I call them and tell them the information on the phone. Then it’s virtual since I don’t see the employee anymore.”

(Interviewee number 23, female, supervisor, *t*_1_)

More specifically, the following problems impeding the management of teams in the A-FO context arising through the physical distance of employees were determined by the interviewees:

*Hampered communication among supervisors and employees*. First, being physically spread within the office seemed to hamper communication not only among team partners, but also between supervisors and team members. This disadvantage was reported by half of the interviewees at *t*_1_ and decreased at *t*_2_ and *t*_3_ (*t*_1_: 50.0%, *t*_2_: 32.0%, *t*_3_: 35.7%). Due to hampered communication, the support and coaching of employees by supervisors have become more challenging, which could be easily done in the previous office environment as illustrated by an interviewee:

“Coaching employees is much more difficult now. In the past, you could simply go to your employee and give him/her advice. Today you have to search for your employee and find a place to talk to him/her.”

(Interviewee number 13, female, supervisor, *t*_1_)

Second, supervisors often complained that they had to tell each piece of information multiple times to reach every team member. In contrast, in the previous office environment, supervisors could simply go to the team and provide the necessary information. Thus, information sharing was reported to be hindered in the A-FO as illustrated by the following example of a supervisor:

“Beforehand, I could simply go into the room where the team members were located and talk to them. Now, it is much more effort to exchange information over the course of the day. Here is a simple example: New Year wishes … Before, I entered the office, said it once and eight people heard it. Now, I have to look for eight individuals or instead I use the impersonal way and just send a mail, which is easier. This is a good example that shows that coordination and effort for me as a supervisor increased – at least if I want to take the more personal way.”

(Interviewee number 13, female, supervisor, *t*_3_)

*Stabilizing the functioning of teams (i.e., team cohesion and information sharing)*. Another challenge that accompanied the physical distance among team members and supervisors was the support of collaboration among team members and ensuring team cohesion, as stated by almost half of the interviewees at all three measurement times (*t*_1_: 50.0%, *t*_2_: 44.0%, *t*_3_: 42.9%). More specifically, supervisors complained that it was challenging to build a team when team members were sitting at different places due to a lack of group dynamic processes, which normally have fostered team cohesion as described by one employee:

“In the old office, the team was also spatially defined. Many things happened informally, which helped to stabilize the functioning of the team. This informal component somehow works like glue that holds team members together. If this is lost because the team is working on a more and more virtual basis, it is more difficult for supervisors to maintain team cohesion.”

(Interviewee number 21, male, employee, *t*_2_)

In connection with these findings, the interviewees mentioned the importance of spending extra effort on team building activities. They highlighted the role of time for informal communication, such as having lunch together so that team partners still felt connected to each other and that supervisors were responsible to arrange such activities. One employee commented:

“The supervisor has to actively work on establishing and maintaining team cohesion. For example, via team-building activities, clear structures through scheduled meetings (jour-fixes) but also via celebrating birthdays and keeping in mind and valuing events that are not directly connected to work itself. That’s the task of supervisors.”

(Interviewee number 11, male, employee, *t*_3_)

Further, interviewees mentioned that regular team meetings were highly relevant in order to ensure information sharing among team partners. For example, one supervisor reported:

“My employees came to me and asked me for more scheduled team meetings since they don’t see each other anymore and don’t know who is working on what. They need a possibility to meet regularly to exchange important information and to keep up to date.”

(Interviewee number 23, female, supervisor, *t*_1_)

*Demand of coordination and time management skills*. Finally, the organization of information sharing within teams, search for employees and rooms, as well as planning team meetings and feedback demanded much coordination and time management skills as alluded to by more than one-third of the interviewees. Importantly, these complaints even increased over the measurement times (*t*_1_: 33.3%, *t*_2_: 36.0%, *t*_3_: 42.9%), suggesting that this was not just a problem due to the change situation *per se* but rather a stable challenge, also after initial routines have been built. One supervisor described the effort it takes to ensure information sharing within teams:

“I offer employees personal meetings if they want, I have introduced scheduled sub team meetings that are obligatory, and I have scheduled meetings with the whole team once a week that are also obligatory. In other words, the A-FO design requires extra time and effort on my part as a supervisor to ensure that team members still meet each other”

(Interviewee number 13, female, supervisor, *t*_2_)

Overall, the interview data suggested that supervisors had to adopt their supervising behaviors to the circumstances arising through the A-FO design. In particular, supervisors and employees reported that supervisors had to deal with the issue that their employees did not necessarily work in their sight and in the sight of their team partners. In line with this, the interviewees reported that supervisors needed to spend extra time in organizing and coordinating information sharing within teams and ensuring team cohesion.

## Discussion

This study explored how teamwork and leadership are affected by an A-FO design. Using a three-wave longitudinal design, this is the first study exploring the impact of A-FOs on teamwork and inter-team collaboration processes over a 2.5-year period. The results suggest potential benefits of A-FO designs for collaboration across teams and between work units, but also demonstrate clear risks for teamwork and the management of teams. With respect to teamwork, we found that A-FO features, such as desk-sharing and activity-related work areas, were reported to negatively affect the physical distance and visibility of team partners. In the A-FO, team partners seemed to be often spread within the office building and thus were less visible to team partners compared to office designs with assigned workstations that were often located close to team members. Consequently, face-to-face communication and collaboration within teams suffered. At first sight, this finding is contradictory to earlier studies on A-FOs having shown that communication and interaction increased when switching to an A-FO (e.g., van der Voordt, 2004; Blok et al., 2009). Yet, these studies did not distinguish between teamwork and inter-team collaboration that might explain the divergent findings. Thus, the results of the current study illustrate the importance of disentangling communication effects for different levels of teamwork. Surprisingly, and in contrast to propositions from the A-FO-M, the interviewed persons did not clearly report that trust among team partners was negatively affected when working in the A-FO. This might be due to long-lasting trusting relationships between the team members in our study prior to switching to the A-FO. Interpersonal trust can be quite stable when it once has been developed and has not been violated (e.g., Lewicki and Bunker, 1995). This assumption is also supported by research on virtual teams. Several studies have demonstrated that initial face-to-face meetings, where team members get to know each other, can help to establish trusting relationships (e.g., Rocco, 1998; Wilson et al., 2006) and thus overcome the risks associated with low visibility and distance among team partners.

Furthermore, we found that A-FO features, in particular desk-sharing and the openness of the work environment, were reported to foster the development of contacts as well as communication across teams due to physical closeness among non-team colleagues. When physical (group) boundaries in A-FOs were reduced compared to traditional office designs, employees from different departments saw each other more often and got in touch more easily. That way, getting familiar with non-team colleagues was eased in A-FOs, which in turn supported the building of trusting relationships. However, although interviewees stated that there was a benefit for inter-team collaboration with respect to contact, communication, and trust, joint project work across teams was reported to having only slightly increased since working in the A-FO. Some interview partners mentioned that such collaborations with non-team colleagues conflicted with individual target agreements, and therefore employees needed to decide whether they would like to spend their time working for their own team or with non-team colleagues. As only the former was listed in their target agreements, they preferred working for their team instead of working with non-team colleagues. These insights show that collaboration across teams in form of joint project work does not simply emerge when working in an A-FO but needs to be specially supported.

In addition, we found that also supervisors were reported having to adopt their leadership behavior to the A-FO design. Supervisors had to spend extra effort on ensuring the functioning of their teams (i.e., organizing and coordinating task accomplishment, organizing information sharing, and ensuring team cohesion), as known from leadership of virtual teams. Still, supervisors and employees are able to meet each other face-to-face in A-FOs, which supervisors reported to make increasing use of by arranging regular face-to-face meetings with their teams. While at *t*_1_ supervisors and employees emphasized that team management in A-FOs resembles the management of virtual teams, this was not clearly mentioned at *t*_3_. Thus, supervisors seemed to have adopted their leadership behaviors to the A-FO context by building new structures to ensure information sharing in teams and team cohesion.

When combining these findings on leadership and the findings on teamwork within teams, where interviewees reported a substantial change from face-to-face communication to electronic media, strong similarities with virtual teams become apparent. This is at least partly surprising, since in A-FOs, employees and supervisors would still have the ability to meet in person in contrast to virtual teams. That is, if supervisors do not actively take counter measures, such as arranging team meetings as mentioned above, the work in A-FOs strongly resembles the work in virtual teams.

Finally, the interview data revealed that most effects remained stable or even increased over time, i.e., they were not only short-term reactions to the new working conditions introduced by the A-FO. One exception is the reported hampered communication among supervisors and employees, which decreased over time, suggesting that persons adapted to the A-FO and developed measures to cope with negative effects.

### Theoretical Implications

This study has several theoretical implications. First, we extend the research on A-FOs by exploring their impact on important team processes, such as information sharing, team cohesion, and trust, which are crucial for team performance but have rarely been investigated so far. The results of our study are in line with a recent theoretical model on A-FOs, the A-FO-M (Wohlers and Hertel, 2017) that theoretically derived how A-FOs affect processes within and across teams.

Second, although existing studies have reported an increase of communication and interaction when switching to an A-FO (van der Voordt, 2004; Blok et al., 2009), few studies have distinguished between teamwork and inter-team collaboration. Our findings revealed diverging effects of A-FO on intra- and inter-team processes with respect to communication and interaction. Hence, this study highlights the importance of distinguishing between these different levels of teamwork, which is consistent with the A-FO-M.

Third, we further extend the research on A-FOs by exploring challenges for the managing of teams in A-FOs. Our findings revealed that supervisors in A-FOs have to face similar challenges as leaders of virtual teams. At the same time, the higher availability of co-located team members in A-FOs compared to geographically distributed members of virtual teams was rarely mentioned as a benefit in the present interviews.

Finally, although the switching to a new office design is a change process, only a few studies have investigated longitudinal effects. However, research on change processes has provided evidence that reactions toward change initiatives vary over the course of time highlighting the importance of exploring the long-term stability of effects as it is possible that immediate reactions change when employees adopt their behavior. Thus, we further contribute to research on A-FOs by demonstrating both effects that are stable and effects that show an evolution over time.

### Practical Implications

With respect to practical implications, this study suggests that A-FOs entail the risk of a disbanding of existing teams whose members still have to work together on specific tasks, and therefore, information exchange and team cohesion should specifically be supported. Team cohesion, for example, can be supported by regular team meetings. A vast amount of research has demonstrated positive effects of regular team meetings on team cohesion and on trust development (e.g., Powell et al., 2004). Hereby, it seems important to not only foster formal, work-related team meetings, such as jour fixes, but also more informal, colloquial meetings, such as going for lunch together or experiencing emotional work events. Such ensure the sharing of personal information, which in particular supports the building of trust and team cohesion (e.g., Zheng et al., 2002). Such measures can help mitigate these risks already known from research on virtual teams. Hence, supervisors should be motivated to spend extra time on ensuring information sharing and cohesion within teams. To mitigate negative effects of the changing situation, organizations should foster such measures already during or even before relocating to an A-FO. Furthermore, it might be useful to test assigning additional team areas (i.e., homebases). That way, team members could still see and meet each other in the office and organizations can still make use of the mixing of the departments. Employees should be trained to be able to choose their work environments as well as their ways of communication according to their personal and their tasks’ needs. For instance, employees should be aware that more complex topics and tasks are better discussed face-to-face in an appropriate surrounding compared to via digital media. In general, a participative leadership approach might be useful in this context, providing employees sufficient autonomy and flexibility to decide where to work, which communication media to use, and how to allocate different subtasks among colleagues and team members.

In addition, this study has demonstrated that joint project work across teams does not simply emerge when working in an A-FO. However, joint project work across teams seems to be a promising extension of teamwork as in this way resources, such as experts, can be used more efficiently. In order to go beyond small talk among non-team colleagues and make use of synergies, it is important to adjust organizational rules and policies, for instance by creating an appropriate code of conduct and adopting target agreements of employees in a way that collaboration across teams is valued and rewarded. Further, the benefit of inter-team collaboration should be communicated to employees and they should be encouraged to proactively initiate joint projects across teams. In summary, this study has demonstrated the importance of adjusting organizational policies, preparing supervisors, and team members for the new circumstances and tasks arising through the A-FO context in order to support the functioning of teams right after switching to the A-FO.

### Limitations and Implications for Future Research

Besides the promising results, this study has some limitations. First, the cooperation partner allowed data collection not before 4 weeks after relocation, unfortunately, so that we only could gather retrospective information about the former office environment and about the relationships between the employees. However, the scope of this article is not a pre-post evaluation of an A-FO design, but an exploration of employees’ subjective reactions to the A-FO relocation over time. Following an explorative approach, no formal hypotheses were tested. Instead, we inductively explored central factors and processes in this respect, for which a qualitative study design can be seen as a valuable approach (e.g., Creswell and Creswell, 2017). Nevertheless, future research is now desirable to validate our findings, for instance, with quasi-experimental interventions in a pre-post design (see benefits of combining qualitative and quantitative research approaches; Creswell and Creswell, 2017). In such follow-up studies, using control groups without new office environments is highly recommended, in order to allow conclusions about causal effects. Nevertheless, in our study, interviewees reported valuable insights on how teamwork and collaboration across teams is affected and changed during working in the A-FO context at each measurement time, allowing comparisons of mentioned effects across time, i.e., exploring the long-term stability of effects.

Second, the sample size of this study is relatively small for all measurement times. Nevertheless, according to literature on qualitative research, a saturation of information typically occurs within the first 12 interviews (Guest et al., 2006). Thus, the number of interviews can be seen as sufficient to address our explorative research questions. In addition, the use of the third wave in the current research design is a benefit even in light of the rather small sample size because this enabled us to examine also non-linear changes in the data pattern which would have been not possible with only two waves (e.g., Singer and Willett, 2003). Moreover, the main reasons for the dropouts in wave 3 (retirement, maternity leave, and job change within the organization) were rather unrelated to the office environment change. Nevertheless, results regarding the data of wave 3 should be interpreted with caution.

Third, this study collected data from only one organization. Given that configurations of A-FOs vary in detail, as well as in the way they are implemented, our results cannot simply be generalized to all other organizational contexts. However, we believe that our findings can be at least partly generalized to organizations that are comparable in regard to the previous office configuration, team structure, and the A-FO configuration (e.g., lacking additional work areas for teams). Future studies might consider data from more than one organization not only to test the generalizability of results but also to consider systematic moderators, for instance, differences in the implementation strategy. Moreover, in order to allow causal conclusions (quasi-)experimental designs are desirable.

Fourth, in the interviews we solely relied on self-reported data due to the interview design. These often raise the concern of common-method-bias (e.g., Podsakoff et al., 2003, 2012). However, self-reports can be sufficient when research is focusing on subjective experiences and perceptions (e.g., Chan, 2009; Conway and Lance, 2010). Moreover, we collected data multiple times to detect changes in communication and interaction behavior in general. Hence, common-method bias due to the use of self-reports can be neglected in this study. Still, future studies addressing team effects in A-FOs should use other sources as well, such as supervisor ratings or some objective data.

Fifth, the reliance on a single interviewer might have biased the results, for example, due to the way questions were asked. To diminish possible interviewer effects, the interviewer practiced the interview protocol with five trained psychologists prior to the interviews in the organization. Moreover, using a single interviewer across waves during the longitudinal study fostered the comparability of contents and results, and also helped to build trust and compliance among the interviewed participants. Therefore, we relied on the first author as the single interviewer.

Additionally, as argued above, we suggest that future research should further explore the impact of different initial states, such as office workers knowing vs. not knowing each other prior to switching to an A-FO, as this might moderate the relationship between switching to an A-FO and team-related processes, such as communication and trust between team members. Experimental designs comparing several initial states seem to be a promising approach. Likewise, the consequences of different A-FO configurations, such as providing an additional area for teams should be investigated. For example, it might be possible that an additional team area would ensure the functioning of teamwork but would also diminish the positive effects shown for inter-team collaboration that seemed to benefit from reduced group boundaries. Quasi-experimental designs might be useful to investigate the impact of these different situations on team outcomes. As A-FO designs vary in detail, this comparison of different situations might reveal how single A-FO features affect teamwork and collaboration across teams, thereby providing valuable insights for researchers and practitioners alike.

Another research topic that we want to stress here is investigating which kind of leadership style seems appropriate in A-FOs. In this study, we only carved out challenges for the management of teams but did not focus on possible benefits for effectively managing teams. Future research should explore such positive effects of A-FOs on leadership. It might be possible that in A-FOs supervisors are forced to change their leadership behavior, since exercising control is less possible in A-FOs due to less visibility of employees (Romeike et al., 2016), in a way that they trust, support, and coach their employees, which has been shown to be positively related to employees’ satisfaction and performance (e.g., Ellinger et al., 2003). Furthermore, besides A-FOs’ working conditions influencing leadership, the other way around is also possible, i.e., leadership might also have an impact on A-FOs’ working conditions and especially how they are perceived by their followers, as addressed in adaptive structuration theory (DeSanctis and Poole, 1994). For example, it might be possible that supervisors insist that employees work in sight of them and close to their team partners, which would considerably impact the above described positive and negative effects of A-FOs on teamwork. Moreover, future studies might systematically investigate the effectiveness of common, well-established leadership approaches in the A-FO context. In particular, studies are needed that shed light on the dynamic leadership and team processes within A-FOs, providing more specific guidelines on effective management strategies in A-FOs. These guidelines should be developed in cooperation with professionals from the organization and researchers from this field. To shed light on the dynamics of A-FOs, teams, and leadership, mixed-method designs using combinations of surveys, observations, or interviews with different sources, supervisors and team members, or quasi-experimental designs, comparing data from teams that can choose workstations on their own vs. are instructed by their supervisors, seem to be promising approaches. Finally, future studies should explore whether research results on virtual leadership can be transferred to the A-FO context (as this study suggested many similarities) or whether team leadership in A-FOs can be conceptualized as a hybrid of face-to-face and virtual leadership, since employees and supervisors can still physically meet each other easily. The results of such studies provide valuable information for organizations on how to optimally support their supervisors in coping with the emerging challenges.

Overall, the findings of this explorative qualitative study can be viewed as a first step in investigating the short- and long-term effects of the A-FO on teamwork, collaboration across teams, and challenges for the management of teams. Next, quantitative, longitudinal studies as well as experimental designs are further needed to support our findings. In addition, it would be useful to also explicitly match team and leadership data as well as to gain objective data on team performance as these are of particular interest for the management who decides on the implementation of A-FOs.

## Conclusion

The trend of redesigning traditional office designs to more flexible, activity-based office designs goes along with alterations of visibility and proximity of team partners, non-team colleagues, as well as supervisors, which critically influence teamwork and team leadership. More specifically, we found that an A-FO design was reported to be beneficial for collaboration across teams, while it hampers teamwork. Along with that, our data suggested that supervisors had to spend extra time and effort to ensure the functioning of their teams. Most effects were reported to remain stable or even increase over time, illustrating that A-FO-related effects might not only be initial reactions to a change initiative but instead are long-term consequences of the design. In order to use A-FOs efficiently, organizations should thus support supervisors and team members to maintain the functioning of teams in such a way that information sharing and cohesion within teams are ensured. Regarding collaboration across teams, organizations should adapt organizational culture and target agreements in a way that joint project work across teams is appreciated and honored.

Together, this explorative study represents a starting point for the exploration of team effects in A-FOs. Future research should now address our findings within a broader context and further validate them using quantitative research approaches.

## Author Contributions

GH arranged the contact to the organization where the interviews were done. CW developed the interview questions, conducted the interviews, developed the coding scheme, coded the material with the help of two independent coders, did the analyses, and wrote the manuscript. During all these stages GH constantly gave advice to CW, gave feedback on the manuscript, and rewrote parts himself.

## Conflict of Interest Statement

The authors declare that the research was conducted in the absence of any commercial or financial relationships that could be construed as a potential conflict of interest.
