# Supplementary material for: Longitudinal Effects of Activity-Based Flexible Office Design on Teamwork
Source: Front Psychol. 2018 Oct 26;9:2016. doi: 10.3389/fpsyg.2018.02016 (PMC6214238; doi:10.3389/fpsyg.2018.02016)
Supplement: Supplementary file 1 [file Table_1.docx]

Supplementary Material

Longitudinal Effects of Activity-based Flexible Office Design on Teamwork

Christina Wohlers*, Guido Hertel

*** Correspondence:** christina.wohlers@uni-muenster.de

# Supplementary Figures and Tables

Appendix A – Description of Functional Work Areas Assigned to Work Activities

| **Work Areas** | **Nature of work activity** | **Individual/group activity** |
| --- | --- | --- |
| **Main Area:** open-layout area containing standard workstations adjustable in height and hot desks, workstations useable for a short period. | All kinds of work activities | Individual |
| **Think Tank**: enclosed space for three to eight persons for concentrated work, short meetings or telephone calls. All think tanks contain a flat screen that can be used for virtual meetings. | Concentrated work | Group |
| **Silent Area**: enclosed space for office workers that need to concentrate on a task for a fixed period. Disturbances (conversation, telephone calls) are forbidden. | Concentrated work | Individual |
| **Project Area**: enclosed space that can be assigned to a temporary project team for a period up to one months if they need to communicate intensively. This area contains moveable workstations. | Creativity / Communication/ Collaboration | Group |
| **Lounge**: open space with an informal, cozy atmosphere containing comfortable seating furniture. For short informal conversations or working alone. | All kinds of work activities | Individual & group |
| **Creative Room:** enclosed space for three to eight persons with an inspirational colorful design and furbishing which should foster creativity. | Creativity/ Communication | Group |
| **Phone Box**: enclosed space for one person. Temporarily useable for spontaneous or planned phone calls. | Communication | Individual |
| **Meeting Room**: enclosed space bookable for formal (planned) meetings. Available for small and large groups and with information technology for virtual meetings. | Communication / Collaboration | Group |
| **Meet & Talk**: open space with an informal, cozy atmosphere containing comfortable seating furniture, coffee facilities and a table football. Zone for informal short communication and relaxation. | All kinds of work activities | Individual & group |
| **Central Archiving**: Central storing space within the office building for all materials. | Services | Individual |
| **Copy & Print**: Centrally located technical area that is visually and acoustically screened off for a distraction-free working environment. | Services | Individual |
| **Lockers**: Centrally located lockers for all office workers to store laptops and personal belongings. | Services | Individual |
| **Postboxes**: Centrally located area with post boxed for every office worker. | Services | Individual |
|  |  |  |

*Note.* Individual activity = working alone; Group activity = working with at least one colleague or team partner.
